# Antibiotics Use for Dental or Oral Cavity Infections in Pediatric Dentistry: Knowledge and Prescribing Practices Between Italian Dentists

**DOI:** 10.3390/antibiotics14040357

**Published:** 2025-03-31

**Authors:** Martina Barone, Michele Basilicata, Giovanni Bruno, Christian Bacci, Patrizio Bollero, Raffaella Docimo, Antonio Gracco, Alberto De Stefani, Filippo Cavallari

**Affiliations:** 1 Department of Neuroscience, School of Dentistry, University of Padova, 35121 Padua, Italy; martina.barone@studenti.unipd.it (M.B.); christian.bacci@unipd.it (C.B.); antonio.gracco@unipd.it (A.G.); alberto.destefani@unipd.it (A.D.S.); filippo.cavallari@unipd.it (F.C.); 2 UOSD Special Care Dentistry, Department of Experimental Medicine and Surgery, University of Roma Tor Vergata, 00133 Rome, Italy; michele.basilicata@ptvonline.it (M.B.); patrizio.bollero@ptvonline.it (P.B.); 3 Department of Biomedical Laboratory Technology, UniCamillus-Saint Camillus International University of Health Sciences, 00133 Rome, Italy; 4 Department of Industrial Engineering, University of Roma Tor Vergata, 00133 Rome, Italy; 5 Pediatric Dentistry, Department of Surgical Sciences, University of Rome Tor Vergata, 00133 Rome, Italy; raffaella.docimo@ptvonline.it; 6 Department of Pharmacological Sciences, University of Padova, 35121 Padua, Italy

**Keywords:** antibiotics, pediatric dentistry, oral infection, dental infection

## Abstract

In pediatric dentistry, antibiotics are currently prescribed for both therapeutic and prophylactic purposes. Antibiotic therapy can be prescribed for the treatment of diffuse dental or oral cavity infections, always as a complement to the most suitable dental procedure for the specific case. The aim of this study was to investigate the knowledge regarding the use and prescribing practices of antibiotics in pediatric patients in a sample of Italian dentists by using an anonymous and telematic questionnaire. Methods: A specially prepared questionnaire was electronically transmitted to a cohort of Italian dentists. The questionnaire consisted of two parts: demographic information and general knowledge of antibiotic prescription in pediatric dentistry. The statistical analysis of the obtained data was performed. Results: The study sample consisted of 242 Italian dentists. Poor statistically significant differences emerged between specialists in Pediatric Dentistry and dentists without specialization or specialists in other branches, as well as between dentists who, in their clinical activity, mainly treat children/adolescents or adults. For the complementary treatment of dental or oral cavity infections, Amoxicillin was the antibiotic indicated as the first choice for pediatric patients with no allergy to penicillins by most of the sample, while more than 20% of dentists would prescribe Clindamycin in patients with an allergy to penicillin. The knowledge regarding the dosage of administration of the chosen antibiotic appeared not to be sufficient. Conclusions: An improvement in the knowledge of the Guidelines in Pediatric Dentistry appeared necessary regarding the posology of the antibiotic of choice. More information about the adverse effects of Clindamycin is needed.

## 1. Introduction

In pediatric dentistry, antibiotics are routinely prescribed and used for both therapeutic and prophylactic purposes [1,2]. Generally, antibiotic therapy is prescribed by the clinician, always as a complement to the most suitable dental procedure for the specific case, for the treatment of diffuse infections of the oral hard and/or soft tissues with the appearance of systemic signs of infection (such as fever higher than 38 °C and facial edema), and in patients severely immunocompromised, given the increased risk of developing complications [3]. Furthermore, antibiotics are prescribed for the prophylaxis of infective endocarditis (IE) in determined categories of patients considered at increased risk of developing IE and at higher risk of adverse outcomes from IE before determined invasive dental procedures, with the purpose of reducing or eliminating the possible transient bacteremia resulting from the latter [4,5].

Since the discovery of penicillin, the use of antibiotics has shown a growing trend in prescriptions among doctors and dentists, resulting in concern about antibiotic resistance, which is a problem of global interest. One of the most frequent causes of the increase in antibiotic use is the lack of knowledge regarding the real indications and need for prescribing them, with consequent excessive and improper use [1,6].

Although in the literature, there are studies on the use of antibiotics in different branches of dentistry (such as oral surgery, implantology, and endodontics) [7,8,9,10,11,12,13], poor literature investigating the knowledge of antibiotic therapy and dentists’ prescribing practices in pediatric dentistry is available, especially in Italy. As a survey allows you to acquire information and data from a representative sample of individuals, using a standardized questionnaire administered in various ways, such as the telematic one, it was decided to perform a questionnaire-based cross-sectional study investigating the knowledge and prescription practices of antibiotic of Italian dentists in pediatric dentistry.

The primary aim of this questionnaire-based cross-sectional study was to investigate the knowledge and practices of antibiotic use in pediatric patients by Italian dentists, both for the complementary treatment of dental or oral cavity infections and for the prophylaxis of infective endocarditis (IE). The secondary aim was to compare these aspects among specialists in pediatric dentistry and general dentists or specialists in other dental branches who treat children. In this article, the authors present the results regarding the use of antibiotics for the complementary treatment of dental or oral cavity infections in pediatric dentistry.

## 2. Results

### 2.1. Study’s Sample Size and Description

The study’s convenience sample consisted of 242 Italian dentists, with a response rate of 34,6%. The statistical analysis revealed that the study sample was prevalently constituted of women (68%), dentists from Northern Italy (70%), dentists who had been practicing dentistry for 20 years or less (around 70%), general dentists (58%), dentists who treat both adults and children/teenagers in their clinical activity (57%) and dentists who practice the profession mainly in the private sector (77%). Table 1 summarizes the characteristics of the study sample.

### 2.2. General Knowledge of the Antibiotics’ Prescription in Pediatric Dentistry

General knowledge of the use of antibiotics in certain clinical situations is reported in Table 2 for the total sample and from Table 3, Table 4, Table 5 and Table 6 on the basis of the sample’s subgroups (i.e., experience, academic level, mainly treated population, sector of dental profession practice).

Regarding the clinical situations in which the prescription of antibiotics is indicated or not, more than 80% of the total sample answered the questions correctly. For the question relating to “Well localized vestibular abscess, with mild or absent facial swelling”, only approximately 50% of the dentists answered correctly.

No statistically significant differences emerged based on experience. However, specialists in pediatric dentistry (*p* = 0.01) and dentists who, in their clinical activity, mainly treat children/teenagers (*p* = 0.03) showed a statistically significant greater preparation for the answer relating to “Replantation of avulsed permanent tooth”. Despite the absence of statistical significance, for “Well-localized vestibular abscess, with little or no facial swelling”, there was a tendency toward greater preparation on the part of specialists in pediatric dentistry, who correctly answered 75%. Statistical significance for “Pulpal or periapical tissue infection, with no clinical signs of systemic infection, in non-medically compromised patient” emerged: dentists who practice the profession only in the private sector showed a higher rate of correctness of the answer compared with those who work only in the public or both public and private sector (*p* = 0.04).

### 2.3. Knowledge of the Antibiotic of First Choice in Pediatric Patients with No Allergy to Penicillins

Knowledge about the antibiotic of first choice for patients with no allergy to penicillins is shown in Table 7 for the total sample and from Table 8, Table 9, Table 10 and Table 11 based on the sample’s subgroups (i.e., experience, academic level, mainly treated population, sector of dental profession practice).

Almost all of the total sample (98%) indicated Amoxicillin as the antibiotic of first choice in pediatric patients with no allergy to penicillins suffering from dental or oral cavity infection. The 85% of the total sample would prescribe the antibiotic therapy for 6 days. No statistically significant differences emerged for the antibiotic of first choice in patients with no allergy to penicillins and related posology based on experience, mainly treated population and sector of dental profession practice. There was a statistically significant greater tendency for general dentists to prescribe the antibiotic for less than 6 days compared with specialists (*p* = 0.01), although the majority of this subgroup sample (>80%) indicated that they would prescribe the antibiotic for 6 days.

### 2.4. Knowledge of the Antibiotic of First Choice in Pediatric Patients with Allergy to Penicillins

Knowledge about the antibiotic of first choice for patients with an allergy to penicillins is shown in Table 12 for the total sample and from Table 13, Table 14, Table 15 and Table 16 based on the sample’s subgroups (i.e., experience, academic level, mainly treated population, sector of dental profession practice).

The majority of the total sample (74%) indicated Macrolide as the antibiotic of first choice in pediatric patients with an allergy to penicillins suffering from dental or oral cavity infection. Only one respondent (0%) indicated Cephalosporin, while 21% would prescribe Clindamycin. Only 18% of the total sample indicated a correct maximum daily dosage of prescription based on the drug of choice; the majority (40%) would prescribe a lower dosage, and 31% would prescribe a higher dosage. Most of the total sample (63%) would prescribe antibiotic therapy for 6 days.

No statistically significant differences emerged for the antibiotic of first choice in patients with an allergy to penicillin and related posology based on experience, academic level, and the mainly treated population. Statistical significance emerged in the antibiotic of first choice based on the sector of dental profession practice (*p* = 0.03): although in all sectors, the antibiotic of first choice was Macrolide, a greater tendency to choose Macrolide was found among dentists who practice the profession only in the private sector if compared with the only public and both public and private sectors; for Clindamycin there was a greater tendency to choose this drug in dentists who practice only in the public sector or both in public and private sector compared with those who practice only in the private sector. As regards posology, no statistically significant differences emerged based on the sector of dental profession practice.

## 3. Discussions

Since their discovery, antibiotics have permitted to treat a wide variety of infections, even deadly ones. However, their correct and responsible use is essential in order to prevent the manifestation of drug-related adverse reactions and to reduce the risk of developing bacterial resistance to the currently available antibiotics. The aim of this study was to investigate Italian dentists’ knowledge and practices of antibiotic use in pediatric patients using a specific questionnaire. In this article, the authors discuss the findings relating to the use of antibiotics for the complementary treatment of dental or oral cavity infections in pediatric dentistry.

In the total sample of 242 Italian dentists, a mean good preparation emerged regarding the clinical situations in which the prescription of antibiotics is indicated or not, as they answered correctly for more than 80% of the questions. According to the current pediatric dentistry available Guidelines of the AAPD [14] and EAPD [15], there are not many oral clinical conditions in which the prescription of antibiotics is really indicated in pediatric dentistry. They can be cited: acute infection, with modest swelling, rapid progression, diffuse cellulitis with moderate-to-severe pain, or with fever (in addition to the treatment of the offending tooth); infection progressed to extraoral fascial spaces; trauma, with significant soft-tissue or dentoalveolar injuries that appear contaminated by debris, extrinsic bacteria or foreign bodies; replantation of avulsed permanent tooth; osteomyelitis; and acute salivary gland swelling of bacterial nature.

It should be noted that to the question relating to “Well-localized vestibular abscess, with little or no facial swelling”, only approximately 50% of dentists answered correctly, and the majority were specialists in pediatric dentistry. According to the mentioned above Guidelines, antibiotic prescription is not indicated in this clinical situation. In fact, endodontic therapy or, if necessary, the extraction of the element responsible for the abscess is often sufficient to resolve the condition in healthy pediatric patients without the need for a complementary antibiotic treatment. However, the prescription of the antibiotic would be indicated (always in addition to dental therapy) if the infection of dental origin shows a tendency toward rapid progression, the manifestation of systemic signs (such as fever), and diffuse cellulitis and/or progression to the extraoral fascial spaces.

In the case of permanent tooth avulsion and subsequent replantation, too, the question in which specialists in pediatric dentistry and dentists who mainly treat children/teenagers in their clinical practice showed a statistically significant greater preparation, the prescription of antibiotic therapy appears indicated. Following a trauma, the periodontal ligament of the avulsed tooth can be contaminated by bacteria present in the oral cavity, in the conservation medium or in the environment in which the avulsion occurred. Therefore, in order to prevent any reactions related to infection and to reduce the risk of inflammatory root resorption, the AAPD and EAPD Guidelines and the Guidelines for the management of dental trauma of the International Association of Dental Traumatology (IADT) [16] recommend the prescription of antibiotic to the patient following reimplantation of the avulsed element, although the role of systemic administration still remains questionable.

According to the Guidelines, in the presence of a pulpal or periapical tissue infection, without clinical signs of systemic infection, in a non-medically compromised patient, the prescription of antibiotics is not indicated. In fact, in case of infection involving the pulpal tissue of the tooth or the tissue immediately surrounding it (i.e., the periapical tissue), systemic antibiotic therapy would not be effective in the resolution of the infection. The indicated treatment is endodontic therapy or the extraction of the involved tooth; a complementary antibiotic therapy would be indicated in the presence of concomitant systemic signs of infection or in immunocompromised patients. For the question relating to this clinical situation, dentists who practice the profession only in the private sector showed a statistically significant higher rate of correctness of the response compared with other sectors of dental profession practice.

As regards to the choice of the most indicated type antibiotic and its relative posology for the complementary pharmacological therapy in pediatric patients suffering from dental or oral cavity infection, the authors of the present study considered a reference the AWaRe Antibiotics Manual [3] (i.e., the Italian translation of the English version of the WHO AWaRe Antibiotic Book of the World Health Organization) published by AIFA, the national authority competent for the regulatory activity of medicines in Italy. According to the Manual, the antibiotic of first choice for the patient with no allergy to penicillin is Amoxicillin, to be assumed orally with a maximum pediatric daily dose of 80–90 mg/kg (to be divided into two equal daily doses, one every 12 h). The Manual indicated the Phenoxymethylpenicillin (or PcV), too; it must be assumed orally with a maximum dosage of 10–15 mg/kg/dose (one every 6–8 h). However, this drug is less used in dentistry in comparison to Amoxicillin. The duration of the antibiotic therapy should be 3 or 5 days, depending on whether the source of the infection has been respectively controlled or not; the patient must be re-evaluated by the dentist before deciding to end the therapy.

Almost all the dentist’s participants of this study correctly indicated Amoxicillin as the first-choice antibiotic in case of dental or oral cavity infection. Nevertheless, most of the total sample would prescribe it with a maximum daily dosage lower than that indicated by AIFA and for an average duration of 6 days. This duration of therapy could be explained by the common and well-known practice among doctors and dentists of prescribing the patient the antibiotic until the end of its packaging (in the case of a tablet formulation); since the package generally contains 12 tablets and the dosage is two tablets per day (one every 12 h), the result is a treatment of 6 days’ duration. The general dentist appeared to be the one who would mostly prescribe antibiotic therapy for less than 6 days compared with the specialist, even though more than 80% of the sample divided based on an academic level, which indicated treatment of 6 days.

True allergy to penicillin, i.e., the immune-mediated allergic reaction (manifested, for example, with anaphylactic shock), is really rare and often self-reported by the patient but not supported by specific diagnostic confirmation tests. According to AIFA’s Manual, in case of true allergy to aminopenicillins (like Amoxicillin or Ampicillin), there is a risk of cross-reaction toward other beta-lactam antibiotics with closely related chemical structures, such as aminocephalosporins (for example, Cephalexin), even if the percentage of allergic patients who can develop allergic reactions if exposed to different beta-lactams is <2% with cephalosporins, <1% with carbapenems and 0% for monobactams. However, in case of severe real allergy to penicillin, an alternative pharmacological option is Macrolides, such as Claritromicin. This antibiotic can be assumed orally, with maximum pediatric daily dosage of 15 mg/kg and a therapy’s duration equal to that of Amoxicillin.

In the present study, most of the total sample indicated Macrolide as the antibiotic of first choice in pediatric patients with an allergy to penicillin suffering from dental or oral cavity infection, while 21% would prescribe Clindamycin. Most of the total sample would prescribe the antibiotic therapy with the incorrect dosage (lower or higher) for 6 days. It must be mentioned that according to the most recent Guidelines of AAPD and AHA [4,5,7,17], Clindamycin has been associated with significant and frequent adverse reactions related to community-acquired Clostridium difficile infections, with possible and even serious complications, including sepsis and death. For this reason, the use of this antibiotic is no longer recommended. In this study, despite the higher general preference for Macrolide, dentists who practice the profession only in the public sector or both in the public and private sector showed a statistically significant greater tendency to choose Clindamycin for allergic patients in comparison with those who practice exclusively in the private sector.

In the literature, similar studies [2,8,18] in pediatric population have been conducted in different countries around the world by different researchers who used specific questionnaires. Levels of adherence to the Guidelines by dentists dealing with pediatric dentistry have been reported variable from 10 to 56%, with rates on average lower among generic dentists if compared to specialists in pediatric dentistry. In general, the reported trend is that of an inappropriate, unjustified, and excessive prescription of antibiotics. In Italy, no other study like this appears to have been conducted in pediatric dentistry. However, similar studies [7,9,10,11,19,20,21,22] have been conducted in the adult population; their results revealed a tendency for excessive and improper use of systemic antibiotics among the interviewed Italian dentists. In fact, it has been reported that many dentists prescribed systemic antibiotics, both for prophylactic and therapeutic purposes, even in clinical situations not recommended by the Guidelines, resulting in over-prescribing. Thus, for both pediatric and adult patients, greater knowledge of the most up-to-date Guidelines between dentists appears necessary.

In the present study, an average good preparation of the total sample emerged in relation to the indications for antibiotic prescription in the pediatric population in case of dental or oral cavity infection, although the authors expected higher levels of preparation, in particular among dentists who are specialists in pediatric dentistry and who mainly treat children/adolescents in their professional activity, given the availability of Guidelines and manuals on this topic. Therefore, a greater knowledge of the Guidelines appears necessary, especially regarding the choice of the type of antibiotic and its posology. This is important for preventing the excessive or inappropriate use of antibiotics and, on a larger scale, to reduce the risk of developing drug-related adverse reactions and bacterial resistance to available antibiotics. Furthermore, more information is needed regarding the adverse effects of Clindamycin, due to which it is no longer recommended by the most recent Guidelines, as a considerable number of Italian dentists would still prescribe this type of antibiotic in pediatric patients with true allergy to penicillin.

The hope of the authors is to have contributed, thanks to the informative material provided to the participants at the end of the compilation of the questionnaire, to diffuse basic knowledge on the correct prescription of antibiotics in pediatric dentistry and to provide the sources through which the dentists will be able to independently inform and stay updated themselves in the future, so that the use of these drugs can become more aware and correct, and unnecessary prescriptions can be drastically reduced.

It should be specified that the sample of specialists in Pediatric Dentistry who participated in the study was limited: only approximately 10% of the total. This limited number can be considered indicative of specialists in this branch currently present in Italy, as the activation of the schools of specialization in pediatric dentistry in Italy is recent and available in fewer than 20 Italian universities. The expectation is that, in Italian specialization schools, importance will be given to the antibiotic therapy in pediatric dentistry by adequately training the residents on the correct prescription of antibiotics. Possible limitations of this study can be represented by the choice to use a convenience sample, the lack of an initial sample size calculation, and the limited number of specialists in pediatric dentistry in the sample.

In addition, a further possible limitation of the study could be the fact that the questionnaire submitted may have appeared excessively long and/or complex, discouraging some participants from continuing until completion and, consequently, the generation of dropouts. This could have negatively influenced the response rate obtained (i.e., 34,6%), which, for the authors, appeared lower than expected: given the response rates reported in similar studies conducted in Italy available in the literature [7,9,10], the expectation was to reach around 50% of responses. However, on the contrary, some participants may have been encouraged to complete the questionnaire with the promise of receiving the final informative material.

It has to be clarified that in the questions where the indication of a duration of prescription of antibiotic therapy was requested, the answer “<6” was considered right; since this value includes all those between 5 and 1, this could appear misleading in the interpretation of data. Although the authors believe that this can have a limited impact on the statistical results of the survey, they consider it correct and transparent to report this clarification as a possible limitation of the present study.

Since the design of this questionnaire-based study could be considered a pre-test one, given the consequent lack of data resulting from a post-test repeated after providing the participants the informative material on antibiotic therapy in pediatric dentistry, the starting point for a future continuation of the study could be represented by the resubmission of the same questionnaire as a post-test to the same cohort of participants, to verify the effectiveness of the informative material provided in terms of professional updating and the propensity for learning of dentists.

Other possible ideas for future research are represented by the repetition of the study when the cohort of specialists in pediatric dentistry will be increased in Italy and by the diffusion of the questionnaire on a larger scale, for example through multicentric studies, conducted in collaboration between various Italian universities.

## 4. Materials and Methods

### 4.1. Questionnaire and Study Sample

A specifically prepared questionnaire was created by using the Google Forms application (Google LLC, Mountain View, CA, USA). The quality of the questionnaire was evaluated as follows. The first version of the questionnaire was ideated and drafted by three authors. Then, this version was administered to other three authors and five dentists operating as tutors of the university dental staff (in a mixed measure of specialists in pediatric dentistry, orthodontics, oral surgery, and general dentistry), who did not previously know its contents; their answers were collected in anonymity, as well as their feedback. Data from this first phase of the study were not included in the final data analysis. Subsequently, the questionnaire, the answers collected and the participants’ feedback were examined by the last two authors, who did not previously know its contents; the aspects assessed were the structure of the questionnaire, clarity and comprehensibility of the language used both in the questions and in the multiple-choice answers, effectiveness of the data collection methods and process, analyzability of the data obtained. Where necessary, questions and answers were reviewed and reformulated.

The final version of the questionnaire was electronically transmitted to a cohort of 700 Italian dentists. Answers were anonymously collected and the consent to use them for research purposes was asked to the participants.

The questionnaire consisted of two parts:

(1) Demographic information—year of birth, sex, experience (i.e., years of dental practices), academic level, mainly treated population, sector of dental profession practice, and geographic Italian region of provenience;
(2) General knowledge of the antibiotics’ prescription in pediatric dentistry—clinical situations in which the use of antibiotics is indicated and practices of use of the latter.

A specially prepared informative material about the prescription of antibiotics in pediatric dentistry was provided to the participants at the end of completing the questionnaire. This material was prepared by the authors; its contents, as well as the correctness of the answers to the questionnaire, were defined on the basis of the Guidelines of the American Academy of Pediatric Dentistry (AAPD) [5,7] and of the European Academy of Pediatric Dentistry (EAPD) [8], the AWaRe Antibiotics Manual of the Agenzia Italiana del Farmaco (AIFA, the national authority competent for the regulatory activity of medicines in Italy, who edited the Italian translation of the English version of the WHO AWaRe Antibiotic Book, published by the World Health Organization) [3] and the American Heart Association (AHA) Guidelines [4,17].

### 4.2. Data Collection

The questionnaire was electronically transmitted to a cohort of 700 Italian dentists. If the participant had not consented to the use of the answers provided for research purposes; the completion of the questionnaire was automatically stopped by the application. For all questions the mandatory answer was set; if an answer to a question had not been provided; the program prevented the completion of the questionnaire from continuing.

Data obtained from the answers given by the participants were organized in a table (Excel, Microsoft Office 365, Microsoft, Remond, WA, USA).

### 4.3. Statistical Analysis

The statistical analysis was performed by the Department of Information Engineering of the University of Padova (Italy). Categorical data were expressed as frequency and percentage, while numerical data were expressed as median and interquartile range (IQR). Categorical data were compared between groups using Fisher’s test and the Chi-square test. No correction for multiple testing was made, as the study was exploratory and not confirmatory. All tests were two-tailed, and a *p*-value < 0.05 was considered statistically significant. Data analysis was performed with R 4.3 (R Foundation for Statistical Computing, Vienna, Austria), REF (R Core Team, 2023. R: A Language and Environment for Statistical Computing. R Foundation for Statistical Computing, Vienna, Austria).

## 5. Conclusions

Although an average good preparation on the prescription of antibiotics in pediatric dentistry emerged, it appeared necessary to improve the knowledge of the guidelines between Italian dentists who deal with children/teenagers’ patients, in particular regarding the type of antibiotic of choice and its posology. Furthermore, more information about the adverse effects of Clindamycin is needed, as many dentists would still prescribe this type of antibiotic.

## Figures and Tables

**Table 1:** Distribution of the sample based on demographic characteristics.

Variable	Category	All (*n* = 242)
Sex	Woman	164 (68%)
	I prefer not to answer	4 (2%)
	Man	74 (31%)
Years of dental practice	10 years or less	92 (38%)
	11–20 years	75 (31%)
	21–30 years	39 (16%)
	More than 30 years	36 (15%)
Academic level	Oral surgery specialist	5 (2%)
	Pediatric dentistry specialist	24 (10%)
	Orthodontics specialist	73 (30%)
	Generic dentist	140 (58%)
Mainly treated population	Adults	32 (13%)
	Adults and children/teenagers	138 (57%)
	Children/teenagers	72 (30%)
Sector of dental profession practice	Private	187 (77%)
	Public	10 (4%)
	Public and private	45 (19%)
Geographic region	North Italy	170 (70%)
	Central Italy	48 (20%)
	South Italy and Islands	24 (10%)

**Table 2:** Right/wrong responses for antibiotic prescription in certain clinical situations in the total sample. The right answer is evidenced in bold.

Variable	Category	All (*n* = 242)
Trauma, with significant soft-tissue or dentoalveolar injuries that appear contaminated by debris, extrinsic bacteria or foreign bodies	No	27 (11%)
	I don’t know	6 (2%)
	**Yes**	209 (86%)
Replantation of avulsed permanent tooth	No	46 (19%)
	I don’t know	12 (5%)
	**Yes**	184 (76%)
Acute infection, with modest swelling, rapid progression, diffuse cellulitis with moderate-to-severe pain, or with fever (in addition to the treatment of the offending tooth)	No	5 (2%)
	**Yes**	237 (98%)
Pulpal or periapical tissue infection, with no clinical signs of systemic infection, in non-medically compromised patient	**No**	202 (83%)
	I don’t know	3 (1%)
	Yes	37 (15%)
Well-localized vestibular abscess, with little or no facial swelling	**No**	128 (53%)
	I don’t know	6 (2%)
	Yes	108 (45%)
Viral infection (for example, primary herpetic gingivostomatitis)	**No**	237 (98%)
	I don’t know	2 (1%)
	Yes	3 (1%)
Infection progressed to extraoral fascial spaces	No	4 (2%)
	I don’t know	2 (1%)
	**Yes**	236 (98%)
Osteomyelitis	No	17 (7%)
	I don’t know	29 (12%)
	**Yes**	196 (81%)
Acute salivary gland swelling of bacterial nature	No	7 (3%)
	I don’t know	10 (4%)
	**Yes**	225 (93%)

**Table 3:** Right/wrong responses for antibiotic prescription in certain clinical situations based on experience (years of dental practice). The right answer is evidenced in bold.

Variable	Category	10 Years or Less (*n* = 92)	11–20 Years (*n* = 75)	21–30 Years (*n* = 39)	More Than 30 Years (*n* = 36)	*p*-Value
Trauma, with significant soft-tissue or dentoalveolar injuries that appear contaminated by debris, extrinsic bacteria or foreign bodies	No	12 (13%)	8 (11%)	4 (10%)	3 (8%)	0.22
	I don’t know	3 (3%)	0 (0%)	3 (8%)	0 (0%)	
	**Yes**	77 (84%)	67 (89%)	32 (82%)	33 (92%)	
Replantation of avulsed permanent tooth	No	16 (17%)	16 (21%)	6 (15%)	8 (22%)	0.94
	I don’t know	5 (5%)	4 (5%)	1 (3%)	2 (6%)	
	**Yes**	71 (77%)	55 (73%)	32 (82%)	26 (72%)	
Acute infection, with modest swelling, rapid progression, diffuse cellulitis with moderate-to-severe pain, or with fever (in addition to the treatment of the offending tooth)	No	3 (3%)	0 (0%)	0 (0%)	2 (6%)	0.15
	**Yes**	89 (97%)	75 (100%)	39 (100%)	34 (94%)	
Pulpal or periapical tissue infection, with no clinical signs of systemic infection, in non-medically compromised patient	**No**	81 (88%)	61 (81%)	34 (87%)	26 (72%)	0.27
	I don’t know	0 (0%)	2 (3%)	0 (0%)	1 (3%)	
	Yes	11 (12%)	12 (16%)	5 (13%)	9 (25%)	
Well-localized vestibular abscess, with little or no facial swelling	**No**	53 (58%)	40 (53%)	18 (46%)	17 (47%)	0.54
	I don’t know	1 (1%)	1 (1%)	2 (5%)	2 (6%)	
	Yes	38 (41%)	34 (45%)	19 (49%)	17 (47%)	
Viral infection (for example, primary herpetic gingivostomatitis)	**No**	91 (99%)	74 (99%)	36 (92%)	36 (100%)	0.17
	I don’t know	0 (0%)	1 (1%)	1 (3%)	0 (0%)	
	Yes	1 (1%)	0 (0%)	2 (5%)	0 (0%)	
Infection progressed to extraoral fascial spaces	No	2 (2%)	0 (0%)	1 (3%)	1 (3%)	0.59
	I don’t know	0 (0%)	1 (1%)	1 (3%)	0 (0%)	
	**Yes**	90 (98%)	74 (99%)	37 (95%)	35 (97%)	
Osteomyelitis	No	10 (11%)	3 (4%)	0 (0%)	4 (11%)	0.22
	I don’t know	13 (14%)	9 (12%)	4 (10%)	3 (8%)	
	**Yes**	69 (75%)	63 (84%)	35 (90%)	29 (81%)	
Acute salivary gland swelling of bacterial nature	No	4 (4%)	1 (1%)	0 (0%)	2 (6%)	0.32
	I don’t know	5 (5%)	2 (3%)	3 (8%)	0 (0%)	
	**Yes**	83 (90%)	72 (96%)	36 (92%)	34 (94%)	

**Table 4:** Right/wrong responses for antibiotic prescription in certain clinical situations based on an academic level. The right answer is evidenced in bold.

Variable	Category	General Dentist (*n* = 141)	Orthodontics or Oral Surgery Specialist (*n* = 77)	Pediatric Dentistry Specialist (*n* = 24)	*p*-Value
Trauma, with significant soft-tissue or dentoalveolar injuries that appear contaminated by debris, extrinsic bacteria or foreign bodies	No	20 (14%)	7 (9%)	0 (0%)	0.24
	I don’t know	4 (3%)	2 (3%)	0 (0%)	
	**Yes**	117 (83%)	68 (88%)	24 (100%)	
Replantation of avulsed permanent tooth	No	36 (26%)	9 (12%)	1 (4%)	**0.01**
	I don’t know	5 (4%)	5 (6%)	2 (8%)	
	**Yes**	100 (71%)	63 (82%)	21 (88%)	
Acute infection, with modest swelling, rapid progression, diffuse cellulitis with moderate-to-severe pain, or with fever (in addition to the treatment of the offending tooth)	No	3 (2%)	2 (3%)	0 (0%)	0.99
	**Yes**	138 (98%)	75 (97%)	24 (100%)	
Pulpal or periapical tissue infection, with no clinical signs of systemic infection, in non-medically compromised patient	**No**	117 (83%)	64 (83%)	21 (88%)	0.48
	I don’t know	1 (1%)	1 (1%)	1 (4%)	
	Yes	23 (16%)	12 (16%)	2 (8%)	
Well-localized vestibular abscess, with little or no facial swelling	**No**	75 (53%)	35 (45%)	18 (75%)	0.13
	I don’t know	3 (2%)	3 (4%)	0 (0%)	
	Yes	63 (45%)	39 (51%)	6 (25%)	
Viral infection (for example, primary herpetic gingivostomatitis)	**No**	137 (97%)	76 (99%)	24 (100%)	0.76
	I don’t know	1 (1%)	1 (1%)	0 (0%)	
	Yes	3 (2%)	0 (0%)	0 (0%)	
Infection progressed to extraoral fascial spaces	No	3 (2%)	1 (1%)	0 (0%)	0.91
	I don’t know	2 (1%)	0 (0%)	0 (0%)	
	**Yes**	136 (96%)	76 (99%)	24 (100%)	
Osteomyelitis	No	8 (6%)	6 (8%)	3 (13%)	0.39
	I don’t know	14 (10%)	11 (14%)	4 (17%)	
	**Yes**	119 (84%)	60 (78%)	17 (71%)	
Acute salivary gland swelling of bacterial nature	No	5 (4%)	0 (0%)	2 (8%)	0.15
	I don’t know	7 (5%)	2 (3%)	1 (4%)	
	**Yes**	129 (91%)	75 (97%)	21 (88%)	

**Table 5:** Right/wrong responses for antibiotic prescription in certain clinical situations based on mainly treated population. The right answer is evidenced in bold.

Variable	Category	Adults (*n* = 32)	Adults and Children/Teenagers (*n* = 138)	Children/Teenagers (*n* = 72)	*p*-Value
Trauma, with significant soft-tissue or dentoalveolar injuries that appear contaminated by debris, extrinsic bacteria or foreign bodies	No	5 (16%)	15 (11%)	7 (10%)	0.78
	I don’t know	1 (3%)	4 (3%)	1 (1%)	
	**Yes**	26 (81%)	119 (86%)	64 (89%)	
Replantation of avulsed permanent tooth	No	12 (38%)	25 (18%)	9 (13%)	**0.03**
	I don’t know	0 (0%)	9 (7%)	3 (4%)	
	**Yes**	20 (63%)	104 (75%)	60 (83%)	
Acute infection, with modest swelling, rapid progression, diffuse cellulitis with moderate-to-severe pain, or with fever (in addition to the treatment of the offending tooth)	No	0 (0%)	3 (2%)	2 (3%)	0.99
	**Yes**	32 (100%)	135 (98%)	70 (97%)	
Pulpal or periapical tissue infection, with no clinical signs of systemic infection, in non-medically compromised patient	**No**	28 (88%)	116 (84%)	58 (81%)	0.75
	I don’t know	0 (0%)	1 (1%)	2 (3%)	
	Yes	4 (13%)	21 (15%)	12 (17%)	
Well-localized vestibular abscess, with little or no facial swelling	**No**	16 (50%)	67 (49%)	45 (63%)	0.19
	I don’t know	1 (3%)	5 (4%)	0 (0%)	
	Yes	15 (47%)	66 (48%)	27 (38%)	
Viral infection (for example, primary herpetic gingivostomatitis)	**No**	32 (100%)	135 (98%)	70 (97%)	0.99
	I don’t know	0 (0%)	1 (1%)	1 (1%)	
	Yes	0 (0%)	2 (1%)	1 (1%)	
Infection progressed to extraoral fascial spaces	No	0 (0%)	4 (3%)	0 (0%)	0.63
	I don’t know	0 (0%)	1 (1%)	1 (1%)	
	**Yes**	32 (100%)	133 (96%)	71 (99%)	
Osteomyelitis	No	0 (0%)	13 (9%)	4 (6%)	0.24
	I don’t know	2 (6%)	19 (14%)	8 (11%)	
	**Yes**	30 (94%)	106 (77%)	60 (83%)	
Acute salivary gland swelling of bacterial nature	No	1 (3%)	4 (3%)	2 (3%)	0.38
	I don’t know	2 (6%)	3 (2%)	5 (7%)	
	**Yes**	29 (91%)	131 (95%)	65 (90%)	

**Table 6:** Right/wrong responses for antibiotic prescription in certain clinical situations based on sector of dental profession practice. The right answer is evidenced in bold.

Variable	Category	Private (*n* = 187)	Public and Public/Private (*n* = 55)	*p*-Value
Trauma, with significant soft-tissue or dentoalveolar injuries that appear contaminated by debris, extrinsic bacteria or foreign bodies	No	21 (11%)	6 (11%)	0.93
	I don’t know	5 (3%)	1 (2%)	
	**Yes**	161 (86%)	48 (87%)	
Replantation of avulsed permanent tooth	No	34 (18%)	12 (22%)	0.51
	I don’t know	8 (4%)	4 (7%)	
	**Yes**	145 (78%)	39 (71%)	
Acute infection, with modest swelling, rapid progression, diffuse cellulitis with moderate-to-severe pain, or with fever (in addition to the treatment of the offending tooth)	No	4 (2%)	1 (2%)	0.99
	**Yes**	183 (98%)	54 (98%)	
Pulpal or periapical tissue infection, with no clinical signs of systemic infection, in non-medically compromised patient	**No**	162 (87%)	40 (73%)	**0.04**
	I don’t know	2 (1%)	1 (2%)	
	Yes	23 (12%)	14 (25%)	
Well-localized vestibular abscess, with little or no facial swelling	**No**	98 (52%)	30 (55%)	0.95
	I don’t know	5 (3%)	1 (2%)	
	Yes	84 (45%)	24 (44%)	
Viral infection (for example, primary herpetic gingivostomatitis)	**No**	184 (98%)	53 (96%)	0.31
	I don’t know	1 (1%)	1 (2%)	
	Yes	2 (1%)	1 (2%)	
Infection progressed to extraoral fascial spaces	No	3 (2%)	1 (2%)	0.99
	I don’t know	2 (1%)	0 (0%)	
	**Yes**	182 (97%)	54 (98%)	
Osteomyelitis	No	10 (5%)	7 (13%)	0.14
	I don’t know	22 (12%)	7 (13%)	
	**Yes**	155 (83%)	41 (75%)	
Acute salivary gland swelling of bacterial nature	No	5 (3%)	2 (4%)	0.89
	I don’t know	8 (4%)	2 (4%)	
	**Yes**	174 (93%)	51 (93%)	

**Table 7:** Knowledge of the antibiotic of first choice for patients with no allergy to penicillins in the total sample. The right answer is evidenced in bold.

Variable	Category	All (*n* = 242)
Antibiotic of first choice in pediatric patient with no allergy to penicillins with odontogenic infection	**Amoxicillin**	237 (98%)
	Clindamycin	2 (1%)
	Macrolide	1 (0%)
	I don’t know	2 (1%)
Maximum daily dosage (considered pediatric weight: <40 kg)	High	15 (6%)
	Low	165 (70%)
	Right	35 (15%)
	I don’t know	22 (9%)
Mean days of prescription (Amoxicillin)	<6 days	27 (11%)
	>6 days	9 (4%)
	6 days	201 (85%)

**Table 8:** Knowledge of the antibiotic of first choice for patients with no allergy to penicillins based on experience (years of dental practice).

Variable	Category	10 Years or Less (*n* = 92)	11–20 Years (*n* = 75)	21–30 Years (*n* = 39)	More Than 30 Years (*n* = 36)	*p*-Value
Antibiotic of first choice in pediatric patient with no allergy to penicillins with odontogenic infection	Amoxicillin	91 (99%)	73 (97%)	38 (97%)	35 (97%)	0.17
	Clindamycin	0 (0%)	2 (3%)	0 (0%)	0 (0%)	
	Macrolide	1 (1%)	0 (0%)	0 (0%)	0 (0%)	
	I don’t know	0 (0%)	0 (0%)	1 (3%)	1 (3%)	
Maximum daily dosage (considered pediatric weight: <40 kg)	High	4 (4%)	4 (5%)	4 (11%)	3 (9%)	0.33
	Low	68 (75%)	52 (71%)	27 (71%)	18 (51%)	
	Right	11 (12%)	12 (16%)	3 (8%)	9 (26%)	
	I don’t know	8 (9%)	5 (7%)	4 (11%)	5 (14%)	
Mean days of prescription (Amoxicillin)	<6 days	9 (10%)	6 (8%)	7 (18%)	5 (14%)	0.26
	>6 days	1 (1%)	3 (4%)	2 (5%)	3 (9%)	
	6 days	81 (89%)	64 (88%)	29 (76%)	27 (77%)	

**Table 9:** Knowledge of the antibiotic of first choice for patients with no allergy to penicillins based on an academic level.

Variable	Category	General Dentist (*n* = 141)	Orthodontics or Oral Surgery Specialist (*n* = 77)	Pediatric Dentistry Specialist (*n* = 24)	*p*-Value
Antibiotic of first choice in pediatric patient with no allergy to penicillins with odontogenic infection	Amoxicillin	136 (96%)	77 (100%)	24 (100%)	0.77
	Clindamycin	2 (1%)	0 (0%)	0 (0%)	
	Macrolide	1 (1%)	0 (0%)	0 (0%)	
	I don’t know	2 (1%)	0 (0%)	0 (0%)	
Maximum daily dosage (considered pediatric weight: <40 kg)	High	10 (7%)	5 (6%)	0 (0%)	0.16
	Low	99 (73%)	50 (65%)	16 (67%)	
	Right	13 (10%)	17 (22%)	5 (21%)	
	I don’t know	14 (10%)	5 (6%)	3 (13%)	
Mean days of prescription (Amoxicillin)	<6 days	21 (15%)	6 (8%)	0 (0%)	**0.01**
	>6 days	2 (1%)	4 (5%)	3 (12%)	
	6 days	113 (84%)	67 (87%)	21 (88%)	

**Table 10:** Knowledge of the antibiotic of first choice for patients with no allergy to penicillins based on mainly treated population.

Variable	Category	Adults (*n* = 32)	Adults and Children/Teenagers (*n* = 138)	Children/Teenagers (*n* = 72)	*p*-Value
Antibiotic of first choice in pediatric patient with no allergy to penicillins with odontogenic infection	Amoxicillin	32 (100%)	133 (96%)	72 (100%)	0.80
	Clindamycin	0 (0%)	2 (1%)	0 (0%)	
	Macrolide	0 (0%)	1 (1%)	0 (0%)	
	I don’t know	0 (0%)	2 (1%)	0 (0%)	
Maximum daily dosage (considered pediatric weight: <40 kg)	High	1 (3%)	10 (8%)	4 (6%)	0.47
	Low	27 (84%)	86 (65%)	52 (72%)	
	Right	2 (6%)	24 (18%)	9 (13%)	
	I don’t know	2 (6%)	13 (10%)	7 (10%)	
Mean days of prescription (Amoxicillin)	<6 days	2 (6%)	21 (16%)	4 (6%)	0.07
	>6 days	0 (0%)	7 (5%)	2 (3%)	
	6 days	30 (94%)	105 (79%)	66 (93%)	

**Table 11:** Knowledge of the antibiotic of first choice for patients with no allergy to penicillins based on sector of dental profession practice. The right answer is evidenced in bold.

Variable	Category	Private (*n* = 187)	Public and Public/Private (*n* = 55)	*p*-Value
Antibiotic of first choice in pediatric patient with no allergy to penicillins with odontogenic infection	**Amoxicillin**	183 (98%)	54 (98%)	0.72
	Clindamycin	2 (1%)	0 (0%)	
	Macrolide	1 (1%)	0 (0%)	
	I don’t know	1 (1%)	1 (2%)	
Maximum daily dosage (considered pediatric weight: <40 kg)	High	10 (5%)	5 (9%)	0.56
	Low	131 (72%)	34 (63%)	
	Right	25 (14%)	10 (19%)	
	I don’t know	17 (9%)	5 (9%)	
Mean days of prescription (Amoxicillin)	<6 days	22 (12%)	5 (9%)	0.25
	>6 days	5 (3%)	4 (7%)	
	6 days	156 (85%)	45 (8%)	

**Table 12:** Knowledge of the antibiotic of first choice for patients with an allergy to penicillins in the total sample.

Variable	Category	All (*n* = 242)
Antibiotic of first choice in pediatric patient with allergy to penicillins with odontogenic infection	Cephalosporin	1 (0%)
	Clindamycin	50 (21%)
	Macrolide	179 (74%)
	I don’t know	8 (3%)
	Tetracycline	4 (2%)
Maximum daily dosage (considered pediatric weight: <40 kg)	High	71 (31%)
	Low	92 (40%)
	Right	41 (18%)
	I don’t know	26 (11%)
Mean days of prescription	<6 days	68 (30%)
	>6 days	12 (5%)
	6 days	146 (63%)
	I don’t know	4 (2%)

**Table 13:** Knowledge of the antibiotic of first choice for patients with no allergy to penicillin based on experience (years of dental practice).

Variable	Category	10 Years or Less (n = 92)	11–20 Years (n = 75)	21–30 Years (n = 39)	More Than 30 Years (n = 36)	*p*-Value
Antibiotic of first choice in pediatric patient with allergy to penicillins with odontogenic infection	Cephalosporin	0 (0%)	0 (0%)	0 (0%)	1 (3%)	0.38
	Clindamycin	26 (28%)	14 (19%)	6 (15%)	4 (11%)	
	Macrolide	62 (67%)	56 (75%)	32 (82%)	29 (81%)	
	I don’t know	3 (3%)	3 (4%)	1 (3%)	1 (3%)	
	Tetracycline	1 (1%)	2 (3%)	0 (0%)	1 (3%)	
Maximum daily dosage (considered pediatric weight: <40 kg)	High	27 (31%)	20 (29%)	13 (34%)	11 (32%)	0.67
	Low	30 (34%)	34 (49%)	13 (34%)	15 (44%)	
	Right	19 (22%)	8 (11%)	8 (21%)	6 (18%)	
	I don’t know	12 (14%)	8 (11%)	4 (11%)	2 (6%)	
Mean days of prescription	<6 days	26 (30%)	23 (33%)	11 (29%)	8 (24%)	0.08
	>6 days	1 (1%)	6 (9%)	1 (3%)	4 (12%)	
	6 days	57 (65%)	41 (59%)	26 (68%)	22 (65%)	
	I don’t know	4 (5%)	0 (0%)	0 (0%)	0 (0%)	

**Table 14:** Knowledge of the antibiotic of first choice for patients with no allergy to penicillin based on an academic level.

Variable	Category	General Dentist (*n* = 141)	Orthodontics or Oral Surgery Specialist (*n* = 77)	Pediatric Dentistry Specialist (*n* = 24)	*p*-Value
Antibiotic of first choice in pediatric patient with allergy to penicillins with odontogenic infection	Cephalosporin	0 (0%)	1 (1%)	0 (0%)	0.11
	Clindamycin	35 (25%)	9 (12%)	6 (25%)	
	Macrolide	96 (68%)	65 (84%)	18 (75%)	
	I don’t know	6 (4%)	2 (3%)	0 (0%)	
	Tetracycline	4 (3%)	0 (0%)	0 (0%)	
Maximum daily dosage (considered pediatric weight: <40 kg)	High	33 (25%)	29 (39%)	9 (38%)	0.12
	Low	56 (43%)	24 (32%)	12 (50%)	
	Right	23 (18%)	16 (21%)	2 (8%)	
	I don’t know	19 (15%)	6 (8%)	1 (4%)	
Mean days of prescription	<6 days	40 (31%)	22 (29%)	6 (25%)	0.21
	>6 days	3 (2%)	7 (9%)	2 (8%)	
	6 days	84 (64%)	46 (61%)	16 (67%)	
	I don’t know	4 (3%)	0 (0%)	0 (0%)	

**Table 15:** Knowledge of the antibiotic of first choice for patients with an allergy to penicillin based on mainly treated population.

Variable	Category	Adults (*n* = 32)	Adults and Children/ Teenagers (*n* = 138)	Children/ Teenagers (*n* = 72)	*p*-Value
Antibiotic of first choice in pediatric patient with allergy to penicillins with odontogenic infection	Cephalosporin	0 (0%)	1 (1%)	0 (0%)	0.97
	Clindamycin	7 (22%)	29 (21%)	14 (19%)	
	Macrolide	24 (75%)	99 (72%)	56 (78%)	
	I don’t know	1 (3%)	6 (4%)	1 (1%)	
	Tetracycline	0 (0%)	3 (2%)	1 (1%)	
Maximum daily dosage (considered pediatric weight: <40 kg)	High	8 (26%)	39 (30%)	24 (34%)	0.90
	Low	14 (45%)	51 (40%)	27 (39%)	
	Right	7 (23%)	23 (18%)	11 (16%)	
	I don’t know	2 (6%)	16 (12%)	8 (11%)	
Mean days of prescription	<6 days	10 (32%)	41 (32%)	17 (24%)	0.69
	>6 days	1 (3%)	7 (5%)	4 (6%)	
	6 days	20 (65%)	79 (61%)	48 (67%)	
	I don’t know	0 (0%)	2 (2%)	3 (4%)	

**Table 16:** Knowledge of the antibiotic of first choice for patients with an allergy to penicillin based on sector of dental profession practice.

Variable	Category	Private (*n* = 187)	Public and Public/Private (*n* = 55)	*p*-Value
Antibiotic of first choice in pediatric patient with allergy to penicillins with odontogenic infection	Cephalosporin	0 (0%)	1 (2%)	**0.03**
	Clindamycin	35 (19%)	15 (27%)	
	Macrolide	145 (78%)	34 (62%)	
	I don’t know	4 (2%)	4 (7%)	
	Tetracycline	3 (2%)	1 (2%)	
Maximum daily dosage (considered pediatric weight: <40 kg)	High	55 (31%)	16 (32%)	0.81
	Low	72 (40%)	20 (40%)	
	Right	34 (19%)	7 (14%)	
	I don’t know	19 (11%)	7 (14%)	
Mean days of prescription	<6 days	53 (29%)	15 (30%)	0.55
	>6 days	10 (6%)	2 (4)	
	6 days	115 (64%)	31 (62%)	
	I don’t know	2 (1%)	2 (4%)	

## Data Availability

Data are available upon request made to the corresponding authors.
